# Foreign Summary

**Published:** 1839-09-07

**Authors:** 


### FOREIGN SUMMARY.

*Lives of celebrated Medical Men.*

BOERHAAVE.—In 1693 he took the degree of Doctor of Physic. His practice at first was very limited, and entirely disproportionate to his ability; but upon the death of Drelincourt, in 1701, by the interest of his friends, he commenced as a lecturer on the institutes of physic; and delivered on this occasion an inaugural discourse—*Oratio de Commendando Studio Hippocratico;* holding forth Hippocrates as the model all students in medicine should adopt. The manner in which he illustrated his discourses by the application of his knowledge in chemistry, induced his pupils, and in particular some English students at Leyden, to request that he would also teach chemistry as he did physic. His fame now began to extend itself, and in 1703, he was invited to a vacant chair of medicine at the University of Groningen, which, however, he declined; and the curators of the Leyden University were so pleased at this conduct, that they issued a decree for an augmentation of his annual salary, and secured to him, when it should become vacant, the chief professorship of medicine. About this time he appears to have first publicly contended for the use of mechanical reasoning in physic; for in 1703 he delivered a discourse, entitled *Oratio de usu ratiocinii in Medicina.* Of the mechanical sect of physicians he may be looked upon as one of the chief supporters; for the doctrines must be regarded to have sprung from Borelli, a native of Naples, born in 1608, and Professor of Philosophy and Mathematics in the Universities of Florence and Pisa. Borelli's work, "De Motu Animalium," gives an account of the functions of animals, which are explained agreeably to the laws of mechanics.

In 1709 Boerhaave succeeded Dr. Hotton as Professor of Medicine and Botany, and in his inaugural discourse, *Oratio quâ repurgatæ Medicinæ facilis asceritur simplicitas*, he exposed the fallacies of the alchemists and metaphysicians, and endeavoured, notwithstanding his predilection for the mechanical and chemical theories, to fix the science of medicine upon the basis of observation, experiment, and the inferences naturally deducible from such a method. His zeal, manifested in the addition of plants connected with medicine, rendered it necessary to extend the Botanic Garden to twice its original dimensions; and such was the esteem felt for him, and the admiration of his labours, that in 1714 he was placed at the head of the University, by receiving the appointment of Rector. In this year, he succeeded the celebrated Bidloo, as Professor of the Practice of Physic, and attended the University Hospital. At this period medicine and surgery were regarded as one and indivisible, and Boerhaave was elected President of the Chirurgical College. In 1718 he succeeded Le Mort as Professor of Chemistry, and gave an oration to prove that chemistry was capable of clearing itself from its own errors. This oration, *De Chemiâ suos Errores expurgante*, laid the foundation of his work on the Elements of Chemistry. In 1721 he delivered an elegant oration on the decease of Professor Bernard Albinus, the father of the celebrated anatomist—*De Vitâ et Obitu Clarrissimi Bernhardi Albini;* and in 1725 he resigned the Rectorship of the University, and pronounced a Discourse on the Method of obtaining Certainty in Physics—*Oratio de comparando certo in Physicis.* This oration subjected him to some inconvenience; for having attacked the doctrine of Des Cartes, the Cartesians were incensed, and concluded that it was thereby intended to introduce scepticism and Spinosism, and that the church would be consequently endangered. The governors of the University called upon Mr. Andala, of Franeker, who had put forth the charge, to retract the opprobrium; this was immediately done, and the means intended to defame the professor, served only to increase his reputation.

In 1728 the Royal Academy of Sciences of Paris, elected him into their body, in the room of the Count Marsigli, deceased; and in 1730 he received a like honour, by admission into the Royal Society of London. In 1729 he was, for the third time, afflicted with gout and severe illness, which compelled him to resign the Professorships of Botany and Chemistry. This act gave rise to an elegant declamation, in which he recounted several particulars of his life. He had been, for a second time, (in 1730,) chosen Rector of the University; and this, in February, 1731, he was obliged to relinquish, as he was no longer able to perform the duties of the office. He delivered on this occasion an oration, *De Honore Medici servitute.* Van Royen succeeded him as professor in the Practical College of Physic, and also in Botany, and Gaubius in Chemistry and the Institutes of Medicine. In 1737 he was attacked with difficulty of breathing, and he described his disease in a letter addressed to Baron Bassand, physician to the Grand Duke of Etruria, which I here subjoin, as it affords an example of the simplicity and clearness of his Latin epistolary style, and also displays the piety of its author:—

"Me prehendit vomica in pulmone, spiritum præfocans ad levissimos corporis motus, a tribus abhinc mensibus quotidie increscens. Si causa augetur, opprimet, si vero rumpitur, eventus incertus. Quicquid fiet, id omne continget ex arbitrio superioris numinis. Cur ego metuam, quid cupiam aliud! Adoremus Deum! sufficit. Interim curo sedulo ut lectissima adhibeam remedia, ut leniam et maturem, securus de exitu. Vixit ultra 68 annos, semperque lætus."

Boerhaave was a very early riser, and regularly devoted many hours to study. He was fond of music, and relieved himself from severer duties by playing on the violin, and singing also. He had a knowledge of music as a science, and had read the principal ancient and modern authors on the subject, as appears from the lectures he delivered on sound and hearing; and he had a concert once a week during the winter, at his residence. "Music is a sweet companion in every stage of life, but to the last it is peculiarly adapted. It furnishes employment without painful exertion, and while it charms the sense, soothes the heart." (*Knox.*) Towards the latter part of his life he frequently retired to his country-seat, and there regaled in an extensive garden, planted with many choice exotics and other treasures of the vegetable kingdom.

His memory was remarkably retentive, a circumstance of great importance to him as a lecturer. He could quote not merely the authors on various topics, but even pages and sections from their works. He enriched his botanical discourses with references to the poets, Ovid, Virgil, and others.

Heberden.—Dr. Heberden's earliest published work was a little pamphlet, entitled ANTIΘHPIAKA, in the form of *An Essay on Mithridatium and Theriaca.* It appeared in 1745, and is characteristic of the precision and accuracy by which the writings of Heberden have been distinguished. The famous antidote to the baneful effects of poisons said to be possessed by Mithridates, king of Pontus, is here shown to have been composed of the most simple ingredients: namely, twenty leaves of rue, one grain of salt, two nuts, and two dried figs. This is given upon the authority of Q. Serenus Samonicus, and is a very different composition to that named after the monarch, in the time of Celsus, which consisted of thirty-eight simples, and these, it appears, were changed every century; so that the Mithridatium of one period bore little or no resemblance to that of another. Dr. Heberden states that the ancients were only acquainted with *three* poisons—hemlock, aconite, and that of venomous beasts, and to these they knew no antidotes. The various effects of different substances may, probably, in the earlier ages, have excited the apprehensions of mankind, and their ignorance of their nature may have given rise to the relation of miraculous stories regarding poisonous substances. Dr. H. very properly ridicules these accounts; such as the concealing of poisons under the stone of a seal or ring, as reported by Theophrastus, or by vapours arising from perfumed gloves or letters. Many modes of poisoning are mentioned as having been practised by the Italians, among others, that of poisoning the clothes. Ben Jonson, in his "Every Man in his Humour," puts the following passage into the mouth of the jealous Kiteley:

"Now, God forbid, O me, now I remember,
My wife drank to me last, and changed the cup;
And bade me wear this CURSED SUIT to-day."

These practices were not confined to Italy. Our own country shared the like, and we learn that Queen Elizabeth was in constant dread of being taken off in this way. Two men were hanged in 1598, for poisoning her saddle! These follies and superstitions are now, thank Heaven, entirely exploded. But the history of the poisoners of the sixteenth century must be read with deep interest.

The Transactions of the Royal College of Physicians, known by the title of "Medical Transactions," were undertaken at the recommendation of Dr. Heberden. He first proposed that the members of the College should collect together their observations, and publish them for the benefit of society. He contributed largely to this design, and his papers constitute some of the most valuable contributions to the work. A very brief preface is attached to this collection of papers. From a sketch, printed in the Appendix to the Commentaries on Diseases, it appears that Dr. Heberden had intended drawing up a very full prefatory address to the Transactions. In this he endeavours to point out the means of improving in natural knowledge, and he shows, that by attentively observing Nature herself, and not building upon the ancients, or upon reasonings *a priori*, a greater progress has been made during the last century than had been till that time from the days of Aristotle. He alludes to the advantages derived from the formation of a common stock by the communications received from all quarters by the learned societies of Europe,—contributions that would never have been made in any other way. The following passage will be read with interest:

"The deference which is sometimes required in physic to the authority of the ancients, would incline any one to suspect that the improvements in the art of healing had not kept pace with those which have been made in other branches of natural knowledge. Philosophers have long ago thrown off Aristotle's tyranny; yet some physicians still choose to wrangle about the meaning of the ancients, rather than to consult Nature herself. Are they afraid of approaching her immediate presence, without making use of the intercession of Hippocrates and Galen? And is that reverence to be still paid to her once faithful ministers, which is properly due to Nature alone, notwithstanding all that Bacon, and Harvey, and Newton, and our other great reformers have witnessed against this mistaken veneration? In works of genius the ancients are unquestionably our superiors and best patterns; but in that sort of knowledge which depends wholly upon experience, the latest writers must in general be the best. Experience may, in politics and morality, be called the teacher of fools; but in the study of nature there is no other guide to true knowledge: accordingly, the practice of physic has been more improved by the casual experiments of illiterate nations, and the rash ones of vagabond quacks, than by the reasonings of all the once celebrated professors of it, and theoretic teachers in the several schools of Europe; very few of whom have furnished us with one new medicine, or have taught us better to use our old ones, or have in any one instance at all improved the art of curing diseases."—*Lancet, from Pettigrew's Medical Portrait Gallery.*

